# Crystal structure of 5,5′-di­bromo-3,3′-di-*tert*-butyl-6,6′-di­methyl­biphenyl-2,2′-diol

**DOI:** 10.1107/S2056989015006313

**Published:** 2015-04-02

**Authors:** Rika Obata, Shigeru Ohba, Yasuaki Einaga, Shigeru Nishiyama

**Affiliations:** a Research and Education Center for Natural Sciences, Keio University, Hiyoshi 4-1-1, Kohoku-ku, Yokohama 223-8521, Japan; b Department of Chemistry, Faculty of Science and Technology, Keio University; and JST-CREST/ACELL, Hiyoshi 3-14-1, Kohoku-ku, Yokohama 223-8522, Japan

**Keywords:** crystal structure, biphen­yl, axial chirality, O—H⋯π inter­actions

## Abstract

The whole mol­ecule of the title compound, C_22_H_28_Br_2_O_2_, is generated by twofold rotation symmetry. The dihedral angle of the biphenyl moiety is 85.05 (11)°. The hy­droxy groups show intra­molecular O—H⋯π inter­actions without any other hydrogen-bond acceptors. In the crystal, there are no other significant inter­molecular inter­actions present.

## Related literature   

For the synthesis of the title compound using a transition-metal catalyst, see: Kubota *et al.* (2012 ▸). For the determination of the absolute configuration of the corresponding (+)-chloro derivative, *viz. S*, see: Gutierrez *et al.* (2010 ▸). For the crystal structure of a similar compound, *i.e.* 5,5′-dimeth­oxy-6,6′-di­methyl­biphenyl-2,2′-diol di­chloro­methane solvate, see: Guo *et al.* (2011 ▸).
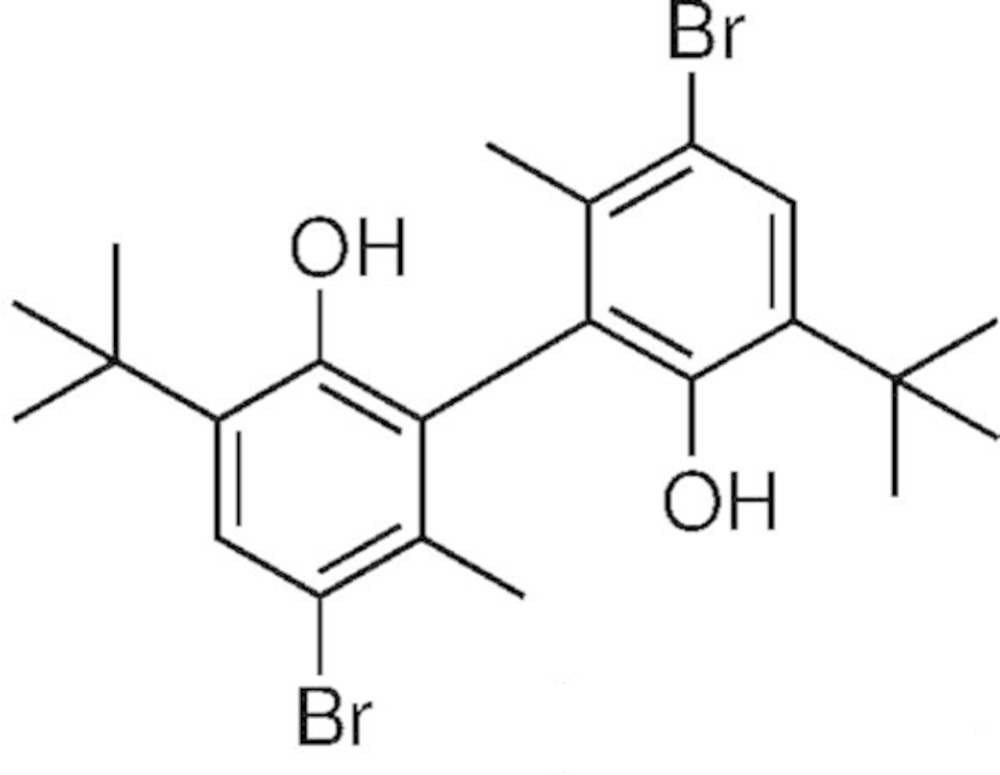


## Experimental   

### Crystal data  

C_22_H_28_Br_2_O_2_
*M* *_r_* = 484.24
Orthorhombic, 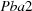

*a* = 7.3680 (5) Å
*b* = 22.4243 (14) Å
*c* = 6.6148 (4) Å
*V* = 1092.91 (12) Å^3^
*Z* = 2
Mo *K*α radiation
μ = 3.72 mm^−1^
*T* = 299 K
0.16 × 0.15 × 0.10 mm

### Data collection  

Bruker D8 VENTURE diffractometer
Absorption correction: multi-scan (*SADABS*; Bruker, 2014 ▸) *T*_min_ = 0.630, *T*_max_ = 0.773
9406 measured reflections
1966 independent reflections
1615 reflections with *I* > 2σ(*I*)
*R*_int_ = 0.036

### Refinement  

*R*[*F* ^2^ > 2σ(*F* ^2^)] = 0.031
*wR*(*F* ^2^) = 0.065
*S* = 1.06
1966 reflections
123 parameters
1 restraint
H-atom parameters constrained
Δρ_max_ = 0.48 e Å^−3^
Δρ_min_ = −0.21 e Å^−3^
Absolute structure: Flack *x* determined using 621 quotients [(*I* ^+^)−(*I* ^−^)]/[(*I* ^+^)+(*I* ^−^)] (Parsons *et al.*, 2013 ▸)
Absolute structure parameter: 0.034 (9)

### 

Data collection: *APEX2* (Bruker, 2014 ▸); cell refinement: *SAINT* (Bruker, 2014 ▸); data reduction: *SAINT*; program(s) used to solve structure: *SHELXS97* (Sheldrick, 2008 ▸); program(s) used to refine structure: *SHELXL2014* (Sheldrick, 2015 ▸); molecular graphics: *SHELXTL* (Sheldrick, 2008 ▸); software used to prepare material for publication: *SHELXL2014* and *publCIF* (Westrip, 2010 ▸).

## Supplementary Material

Crystal structure: contains datablock(s) global, I. DOI: 10.1107/S2056989015006313/su5104sup1.cif

Structure factors: contains datablock(s) I. DOI: 10.1107/S2056989015006313/su5104Isup2.hkl

Supporting information file. DOI: 10.1107/S2056989015006313/su5104Isup3.cml

**Fig. 1.**
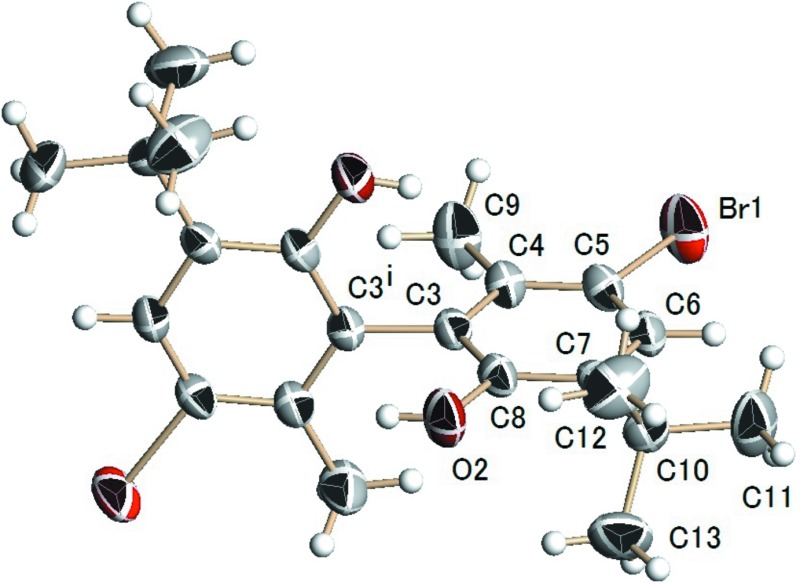
The molecular structure of the title compound, with atom labelling. Displacement ellipsoids are drawn at the 50% probability level.

. DOI: 10.1107/S2056989015006313/su5104fig1.tif
The mol­ecular structure of the title compound, with atom labelling. Displacement ellipsoids are drawn at the 50% probability level.

**Fig. 2.**
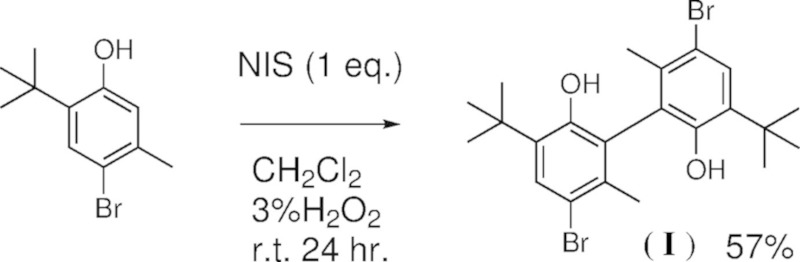
The synthesis of the title compound, (I).

. DOI: 10.1107/S2056989015006313/su5104fig2.tif
The synthesis of the title compound, (I).

CCDC reference: 1056738

Additional supporting information:  crystallographic information; 3D view; checkCIF report

## Figures and Tables

**Table 1:** Hydrogen-bond geometry (, ) *Cg* is the centroid of benzene ring C3C8.

*D*H*A*	*D*H	H*A*	*D* *A*	*D*H*A*
O2H2*Cg* ^i^	0.82	2.54	3.047(5)	122

Symmetry code: (i) 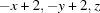.

## supplementary crystallographic information

### S1. Synthesis and crystallization

The synthesis of the title compound, (I), is described in Fig. 2. It was prepared using iodine-mediated coupling method from 4-bromo-2-*tert*-butyl-5-methyl­phenol. To the solution of 4-bromo-2-*tert*-butyl-5-methyl­phenol (0.242 g, 1 mmol) in di­chloro­methane (1 mL) was added *N*-iodo­succinimide (abbreviated to NIS, 0.225 g, 1 mmol) and 3% H_2_O_2_ (1 mL). After shaking (200 rpm) the reaction mixture for 24 h at room temperature, it was poured into saturated Na_2_S_2_O_3_ solution, and extracted with chloro­form. The organic layer was washed with saturated NaCl and dried over anhydrous Na_2_SO_4_. The mixture was evaporated and purified by silica-gel column chromatography to give title compound (I) as white solid (yield: 0.138 g, 57%). ^1^H-NMR (400 MHz, CDCl_3_) 1.32 (18H, s), 1.92 (6H, s), 4.80 (2H, s), 7.47 (2H, s). Tof-MS ES(-) Anal. 481.0357, Calcd. 481.0378 for C_22_H_27_O_2_Br_2_. The crystals were grown by slow evaporation from a toluene/*n*-hexane (1/4) solution.

### S2. Refinement

Crystal data, data collection and structure refinement details are summarized in the experimental table. The hydroxyl H atom was located from a difference Fourier map but was refined as riding (AFIX 147) with *U*_iso_(H) = 1.5*U*_eq_(O). C-Bound H atoms were included in calculated positions and refined as riding: C—H = 0.93–0.96 Å with *U*_iso_(H) = 1.5*U*_eq_(C) for methyl H atoms and 1.2*U*_eq_(C) for other H atoms.

### Crystal data

**Table:**

C_22_H_28_Br_2_O_2_	*D*_x_ = 1.472 Mg m^−^^3^
*M**_r_* = 484.24	Mo *K*α radiation, λ = 0.71073 Å
Orthorhombic, *P**b**a*2	Cell parameters from 4121 reflections
*a* = 7.3680 (5) Å	θ = 2.9–23.6°
*b* = 22.4243 (14) Å	µ = 3.72 mm^−^^1^
*c* = 6.6148 (4) Å	*T* = 299 K
*V* = 1092.91 (12) Å^3^	Prism, colourless
*Z* = 2	0.16 × 0.15 × 0.10 mm
*F*(000) = 492	

### Data collection

**Table:**

Bruker D8 VENTURE diffractometer	1615 reflections with *I* > 2σ(*I*)
Radiation source: fine-focus sealed tube	*R*_int_ = 0.036
ω scans	θ_max_ = 25.3°, θ_min_ = 2.9°
Absorption correction: multi-scan (*SADABS*; Bruker, 2014)	*h* = −8→8
*T*_min_ = 0.630, *T*_max_ = 0.773	*k* = −26→26
9406 measured reflections	*l* = −7→7
1966 independent reflections	

### Refinement

**Table:**

Refinement on *F*^2^	Hydrogen site location: inferred from neighbouring sites
Least-squares matrix: full	H-atom parameters constrained
*R*[*F*^2^ > 2σ(*F*^2^)] = 0.031	*w* = 1/[σ^2^(*F*_o_^2^) + (0.0176*P*)^2^ + 0.3778*P*] where *P* = (*F*_o_^2^ + 2*F*_c_^2^)/3
*wR*(*F*^2^) = 0.065	(Δ/σ)_max_ = 0.001
*S* = 1.06	Δρ_max_ = 0.48 e Å^−^^3^
1966 reflections	Δρ_min_ = −0.21 e Å^−^^3^
123 parameters	Absolute structure: Flack *x* determined using 621 quotients [(*I*^+^)-(*I*^-^)]/[(*I*^+^)+(*I*^-^)] (Parsons *et al.*, 2013)
1 restraint	Absolute structure parameter: 0.034 (9)
Primary atom site location: structure-invariant direct methods	

### Special details

**Table:**

Geometry. All e.s.d.'s (except the e.s.d. in the dihedral angle between two l.s. planes) are estimated using the full covariance matrix. The cell e.s.d.'s are taken into account individually in the estimation of e.s.d.'s in distances, angles and torsion angles; correlations between e.s.d.'s in cell parameters are only used when they are defined by crystal symmetry. An approximate (isotropic) treatment of cell e.s.d.'s is used for estimating e.s.d.'s involving l.s. planes.

### Fractional atomic coordinates and isotropic or equivalent isotropic displacement parameters (Å^2^)

**Table:**

	*x*	*y*	*z*	*U*_iso_*/*U*_eq_	
Br1	1.18668 (8)	0.81211 (2)	0.48288 (15)	0.0681 (2)	
O2	0.7528 (4)	0.98204 (13)	0.9897 (9)	0.0576 (8)	
H2	0.7777	1.0166	0.9607	0.086*	
C3	0.9856 (6)	0.96676 (17)	0.7429 (7)	0.0352 (10)	
C4	1.0903 (6)	0.9290 (2)	0.6223 (7)	0.0388 (11)	
C5	1.0536 (6)	0.8689 (2)	0.6397 (7)	0.0399 (11)	
C6	0.9212 (6)	0.84644 (19)	0.7649 (7)	0.0394 (11)	
H6	0.9023	0.8054	0.7685	0.047*	
C7	0.8150 (6)	0.88292 (19)	0.8859 (7)	0.0360 (10)	
C8	0.8518 (6)	0.94415 (19)	0.8707 (7)	0.0370 (11)	
C9	1.2322 (7)	0.9538 (2)	0.4852 (15)	0.0650 (14)	
H9A	1.1993	0.9460	0.3473	0.098*	
H9B	1.3467	0.9353	0.5143	0.098*	
H9C	1.2417	0.9960	0.5058	0.098*	
C10	0.6668 (5)	0.8582 (2)	1.0234 (8)	0.0428 (15)	
C11	0.6564 (7)	0.7905 (2)	1.0111 (14)	0.074 (2)	
H11A	0.6243	0.7789	0.8761	0.111*	
H11B	0.5661	0.7762	1.1039	0.111*	
H11C	0.7722	0.7737	1.0456	0.111*	
C12	0.7053 (8)	0.8746 (3)	1.2449 (9)	0.0756 (18)	
H12A	0.8260	0.8623	1.2802	0.113*	
H12B	0.6195	0.8548	1.3311	0.113*	
H12C	0.6944	0.9170	1.2619	0.113*	
C13	0.4827 (6)	0.8829 (2)	0.9625 (15)	0.0760 (17)	
H13A	0.4791	0.9249	0.9894	0.114*	
H13B	0.3893	0.8632	1.0387	0.114*	
H13C	0.4634	0.8760	0.8208	0.114*	

### Atomic displacement parameters (Å^2^)

**Table:**

	*U*^11^	*U*^22^	*U*^33^	*U*^12^	*U*^13^	*U*^23^
Br1	0.0866 (4)	0.0458 (3)	0.0718 (4)	0.0118 (2)	0.0311 (4)	−0.0118 (4)
O2	0.0646 (19)	0.0366 (16)	0.072 (2)	0.0049 (14)	0.018 (3)	−0.009 (3)
C3	0.040 (3)	0.030 (2)	0.036 (3)	0.0016 (19)	−0.004 (2)	0.001 (2)
C4	0.048 (3)	0.033 (3)	0.035 (3)	0.003 (2)	0.002 (2)	0.000 (2)
C5	0.048 (3)	0.035 (3)	0.037 (3)	0.009 (2)	0.003 (2)	−0.003 (2)
C6	0.046 (3)	0.028 (2)	0.045 (3)	0.002 (2)	−0.002 (2)	−0.002 (2)
C7	0.036 (3)	0.034 (2)	0.038 (2)	0.008 (2)	−0.005 (2)	−0.002 (2)
C8	0.042 (3)	0.031 (3)	0.038 (2)	0.006 (2)	0.000 (2)	−0.005 (2)
C9	0.084 (3)	0.048 (3)	0.063 (3)	0.003 (2)	0.033 (5)	−0.003 (5)
C10	0.035 (3)	0.045 (3)	0.048 (4)	−0.0010 (19)	0.008 (3)	0.003 (2)
C11	0.070 (3)	0.048 (3)	0.104 (6)	−0.013 (2)	0.036 (5)	0.008 (4)
C12	0.078 (4)	0.099 (5)	0.049 (4)	−0.021 (4)	0.010 (3)	0.002 (3)
C13	0.039 (3)	0.086 (4)	0.103 (5)	0.005 (2)	0.012 (4)	0.020 (5)

### Geometric parameters (Å, º)

**Table:**

Br1—C5	1.913 (4)	C9—H9B	0.9600
O2—C8	1.369 (6)	C9—H9C	0.9600
O2—H2	0.8200	C10—C13	1.520 (7)
C3—C8	1.394 (6)	C10—C11	1.521 (7)
C3—C4	1.396 (6)	C10—C12	1.537 (8)
C3—C3^i^	1.506 (8)	C11—H11A	0.9600
C4—C5	1.379 (6)	C11—H11B	0.9600
C4—C9	1.491 (8)	C11—H11C	0.9600
C5—C6	1.375 (6)	C12—H12A	0.9600
C6—C7	1.386 (6)	C12—H12B	0.9600
C6—H6	0.9300	C12—H12C	0.9600
C7—C8	1.403 (6)	C13—H13A	0.9600
C7—C10	1.526 (6)	C13—H13B	0.9600
C9—H9A	0.9600	C13—H13C	0.9600
			
C8—O2—H2	109.5	C13—C10—C11	107.7 (4)
C8—C3—C4	121.1 (4)	C13—C10—C7	110.4 (5)
C8—C3—C3^i^	117.3 (4)	C11—C10—C7	111.5 (4)
C4—C3—C3^i^	121.6 (4)	C13—C10—C12	109.3 (5)
C5—C4—C3	115.9 (4)	C11—C10—C12	107.4 (5)
C5—C4—C9	123.5 (4)	C7—C10—C12	110.5 (4)
C3—C4—C9	120.5 (4)	C10—C11—H11A	109.5
C6—C5—C4	123.2 (4)	C10—C11—H11B	109.5
C6—C5—Br1	116.5 (3)	H11A—C11—H11B	109.5
C4—C5—Br1	120.3 (3)	C10—C11—H11C	109.5
C5—C6—C7	122.2 (4)	H11A—C11—H11C	109.5
C5—C6—H6	118.9	H11B—C11—H11C	109.5
C7—C6—H6	118.9	C10—C12—H12A	109.5
C6—C7—C8	115.3 (4)	C10—C12—H12B	109.5
C6—C7—C10	122.2 (4)	H12A—C12—H12B	109.5
C8—C7—C10	122.5 (4)	C10—C12—H12C	109.5
O2—C8—C3	120.0 (4)	H12A—C12—H12C	109.5
O2—C8—C7	117.6 (4)	H12B—C12—H12C	109.5
C3—C8—C7	122.4 (4)	C10—C13—H13A	109.5
C4—C9—H9A	109.5	C10—C13—H13B	109.5
C4—C9—H9B	109.5	H13A—C13—H13B	109.5
H9A—C9—H9B	109.5	C10—C13—H13C	109.5
C4—C9—H9C	109.5	H13A—C13—H13C	109.5
H9A—C9—H9C	109.5	H13B—C13—H13C	109.5
H9B—C9—H9C	109.5		
			
C8—C3—C4—C5	−0.5 (6)	C3^i^—C3—C8—O2	0.0 (6)
C3^i^—C3—C4—C5	178.5 (4)	C4—C3—C8—C7	0.1 (7)
C8—C3—C4—C9	179.6 (5)	C3^i^—C3—C8—C7	−179.0 (4)
C3^i^—C3—C4—C9	−1.4 (7)	C6—C7—C8—O2	−178.9 (4)
C3—C4—C5—C6	0.9 (7)	C10—C7—C8—O2	1.9 (6)
C9—C4—C5—C6	−179.3 (6)	C6—C7—C8—C3	0.1 (6)
C3—C4—C5—Br1	179.9 (3)	C10—C7—C8—C3	−179.1 (4)
C9—C4—C5—Br1	−0.2 (7)	C6—C7—C10—C13	−118.6 (5)
C4—C5—C6—C7	−0.8 (7)	C8—C7—C10—C13	60.5 (6)
Br1—C5—C6—C7	−179.8 (3)	C6—C7—C10—C11	1.0 (6)
C5—C6—C7—C8	0.3 (7)	C8—C7—C10—C11	−179.8 (5)
C5—C6—C7—C10	179.4 (4)	C6—C7—C10—C12	120.4 (5)
C4—C3—C8—O2	179.0 (4)	C8—C7—C10—C12	−60.5 (6)

Symmetry code: (i) −*x*+2, −*y*+2, *z*.

### Hydrogen-bond geometry (Å, º)

Cg is the centroid of benzene ring C3–C8.

**Table:**

*D*—H···*A*	*D*—H	H···*A*	*D*···*A*	*D*—H···*A*
O2—H2···*Cg*^i^	0.82	2.54	3.047 (5)	122

Symmetry code: (i) −*x*+2, −*y*+2, *z*.
