# Supplementary figures and images for: Crystal structure of 5,5′-di­bromo-3,3′-di-tert-butyl-6,6′-di­methyl­biphenyl-2,2′-diol
Source: Acta Crystallogr E Crystallogr Commun. 2015 Apr 2;71(Pt 5):o278–9. doi: 10.1107/S2056989015006313 (PMC4420096; doi:10.1107/S2056989015006313)

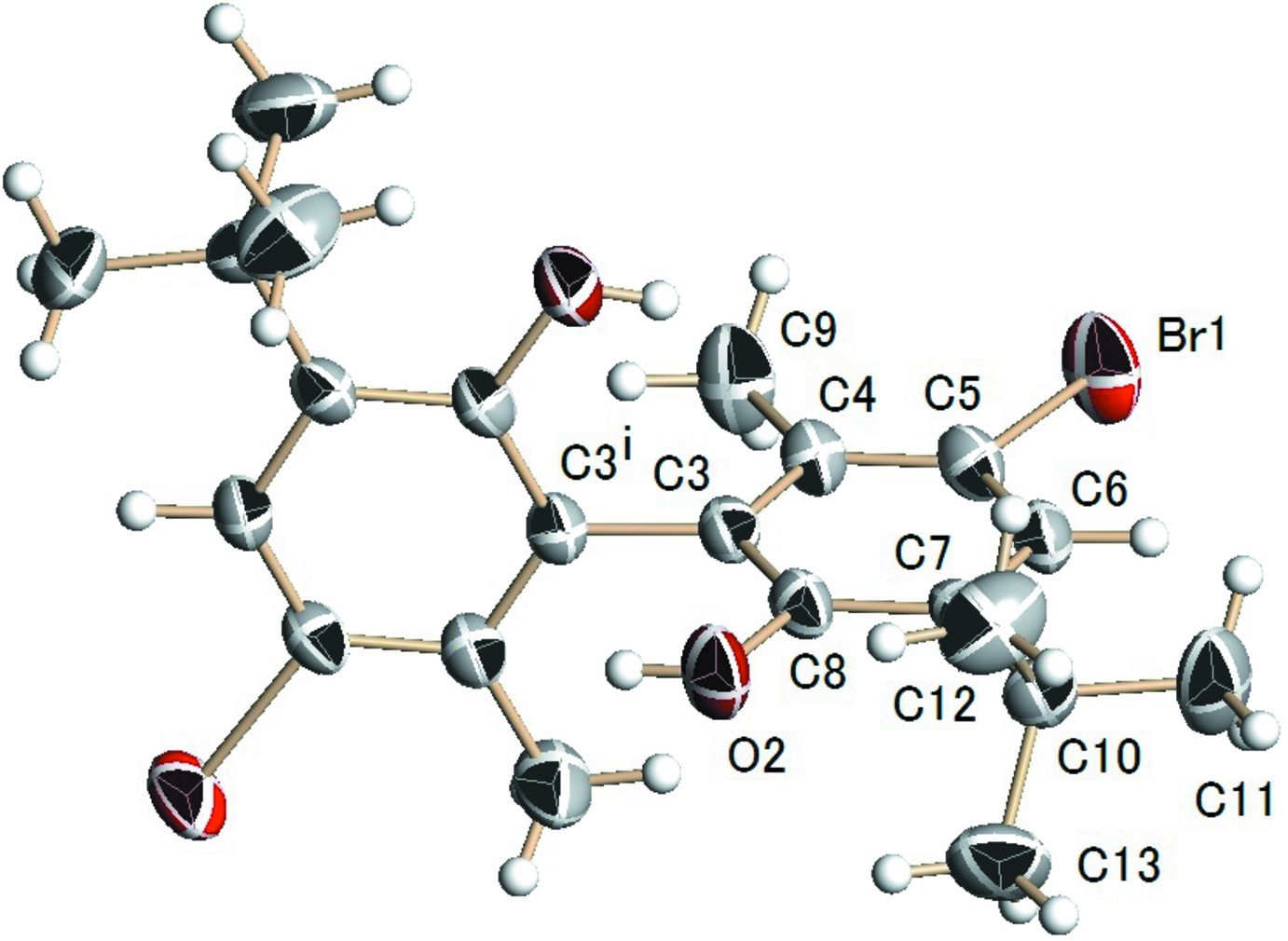

Supplement: Supplementary file 4 [file e-71-0o278-fig1.tif]

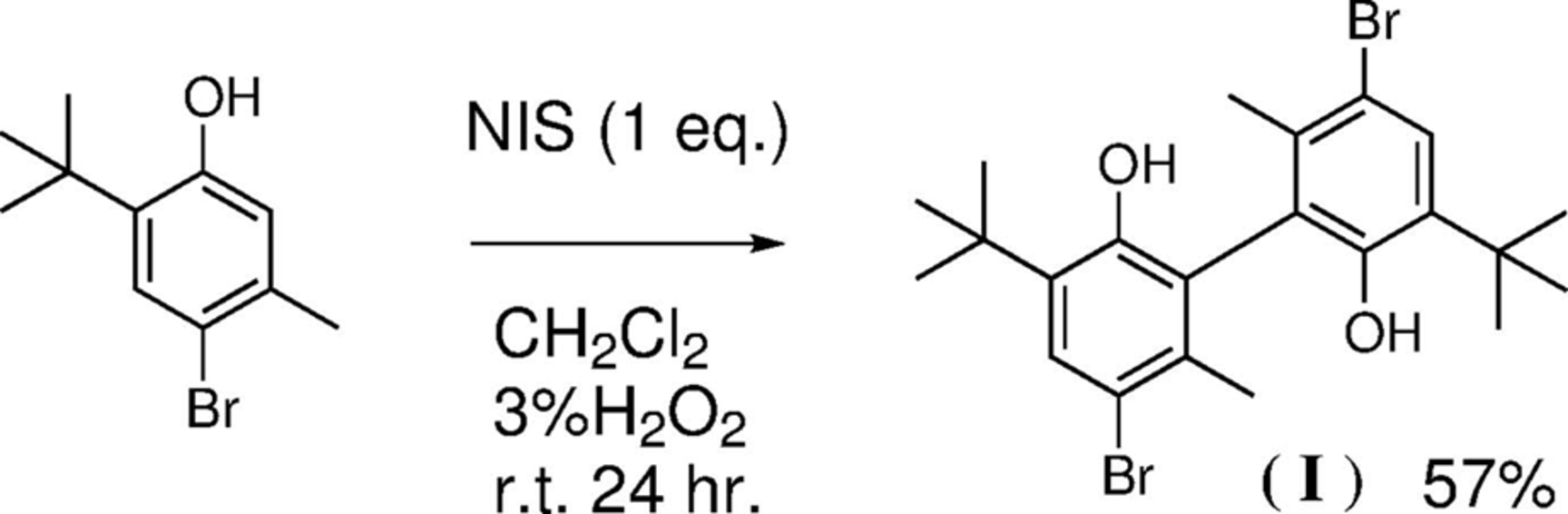

Supplement: Supplementary file 5 [file e-71-0o278-fig2.tif]
